# AXL tyrosine kinase inhibitor TP-0903 induces ROS trigger neuroblastoma cell apoptosis via targeting the miR-335-3p/DKK1 expression

**DOI:** 10.1038/s41420-025-02681-9

**Published:** 2025-08-13

**Authors:** Tsai-Yi Tseng, Shao-Hsuan Kao, Shun-Fa Yang, Yi-Chen Lin, Chu-Liang Lin, Juei-Liang Chen, Chien-Min Chen, Yi-Hsien Hsieh

**Affiliations:** 1 Division of Pediatric Surgery, Department of Surgery, Children’s Hospital of China Medical University, Taichung, Taiwan; 2 Institute of Medicine, Chung Shan Medical University, Taichung, Taiwan; 3 Department of Medical Research, Chung Shan Medical University Hospital, Taichung, Taiwan; 4 Department of Pathology & Laboratory Medicine, Taichung Veterans General Hospital, Taichung, Taiwan; 5 Division of Neurosurgery, Department of Surgery, Changhua Christian Hospital, Changhua, Taiwan; 6 Department of Post-Baccalaureate Medicine, College of Medicine, National Chung Hsing University, Taichung, Taiwan; 7 Department of Biomedical Sciences National Chung Cheng University, Chiayi, Taiwan

**Keywords:** Apoptosis, RNAi, miRNAs

## Abstract

Neuroblastoma (NB) is an aggressive cancer and has poor prognosis in children. TP-0903, a multi-kinase inhibitor, shows inhibitory effects on NB but the mechanistic act is not completely explored. Here, we aimed to explore the anticancer activity of TP-0903 against NB cells and its underlying mechanism. In this study, our findings showed that TP-0903 ( ≥ 50 nM) significantly inhibited the growth of SH-SY5Y and Neuro-2a cells. Further results revealed that TP-0903 remarkably triggered cell apoptosis, mitochondrial membrane potential (MMP) lose, and caspase activation. Microarray assay, qRT-PCR, and Western blotting results indicated that DKK1 was downregulated by TP-0903. Notably, DKK1 is upregulated in NB tissues as comparing to normal tissues. Moreover, silencing DKK1 promoted TP-0903-induced apoptosis and caspase activation, and predicted the binding of TP-0903 to DKK1. In addition, we found that 3’-UTR of DKK1 had a potential target region for miR-335-3p and TP-0903 upregulated miR-335-3p expression. Of important, miR-335-3p mimic combined with TP-0903 provoked higher apoptosis and caspase activation than TP-0903 alone. We also observed that TP-0903 increased cellular reactive oxygen species (ROS), and inhibition of ROS reduced the apoptosis, PARP cleavage, and miR-335-3p, while increasing DKK1 in response to TP-0903. Finally, we demonstrated that TP-0903 significantly diminished the tumor growth and DKK1 expression in xenograft mice. Collectively, our findings indicate that TP-0903 triggers apoptotic cell death of NB cells, attributing to the ROS-mediated miR-335-3p upregulation and the consequent DKK1 downregulation.

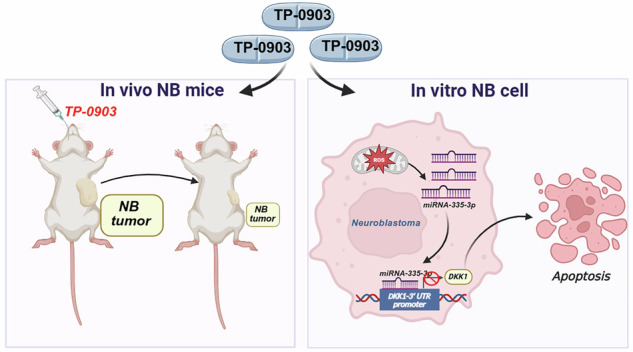

## Introduction

Neuroblastoma is a malignant tumor that originates from the neural crest cells of the sympathetic nervous system and is a leading cause of cancer-related deaths in children [1]. This type of cancer primarily develops in the adrenal glands and paraspinal ganglia, but it can also spread to other areas, commonly affecting the bones, lymph nodes, liver, and bone marrow [2]. Although some NB lesions may regress spontaneously, the disease is highly aggressive, and many patients ultimately succumb to recurrent, refractory, or metastatic disease [3]. Children diagnosed with metastatic NB often present with symptoms such as fever, bone pain, and weight loss Treatment is typically staged and stratified by risk, with certain aspects of radiation therapy adjusted based on the patient’s response. Current treatment strategies for NB include a combination of surgery, chemotherapy with autologous stem cell rescue, radiation therapy, and, in some cases, immunotherapy [4]. However, most children with NB experience long-term side effects that affect their development and quality of life [5].

Protein tyrosine kinases (PTKs) and the associated pathways are crucial in regulating tumorigenic features such as proliferation, invasion, and acquired drug resistance [6]. TP-0903 is an ATP-competitive inhibitor that features an adenine-mimicking heterocyclic moiety, allowing it to bind effectively to the active conformation of AXL [7], and has gained attention for its promising anticancer potential, particularly in cancers with dysregulated signaling pathways [8–10]. Additionally, TP-0903 targets other kinases, including all three members of the TAM family (Tyro3, AXL, and MerTK), as well as Aurora A, JAK2, ALK, ABL1, and VEGFR2 [11]. A prior study indicates that TP-0903 and its combination with conventional anticancer drugs demonstrate inhibitory effects on NB cells, which are linked to the induction of apoptosis and cell cycle arrest [12]. However, the detailed mechanisms through which TP-0903 inhibits the growth of NB cells have not been fully explored.

Dickkopf1 (DKK1), first discovered as a factor that promotes head formation during embryonic development, plays a crucial role in embryogenesis [13]. In tumorigenesis, DKK1 functions as a tumor suppressor through its inhibition of Wnt signaling [14]. However, DKK1 upregulation has also been associated with poor prognosis in rectal cancer, non-small cell lung cancer, and esophageal squamous cell carcinoma [15, 16]. Recent studies reported that upregulation of miR-335-3p exerts anticancer potentials, including inhibition of cell proliferation and induction of cell cycle arrest that attribute to targeting EIF3E and BCL-W and SP1 in acute myeloid leukemia cell [17], and induction of apoptosis in lung cancer cell [18]. In high glucose-induced osteoblast apoptosis, miR-335-3p is downregulated while DDK1 is upregulated, and reversely, overexpression of miR-335-3p decreases DKK1, suggesting that miR-335-3p targets DKK1 and subsequently modulates the apoptosis of osteoblast [19]. Nevertheless, the role and interlink between miR-335-3p and DKK1 in neuroblastoma cells have received minimal investigation.

In this study, we aimed to explore the anti-NB activity of TP-0903 and the underlying mechanistic actions. Our findings indicate that TP-0903 leads to mitochondrial membrane potential (MMP) loss and reactive oxygen species (ROS) elevation. RNA-seq analysis reveals that TP-0903 regulates a spectrum of gene expression, which are involved various biological processes. Further investigation reveals that TP-0903 triggers apoptosis of NB cells through multiple pathways including direct interaction with DKK1, microRNA (miRNA)-mediated DKK1 downregulation, and ROS-mediated miRNA upregulation. Furthermore, in vivo assessment was also conducted to support the mechanistic actions induced by TP-0903. These findings not only unveil the anti-NB mechanism triggered by TP-0903, but also provide further evidence to support the anti-NB therapeutic potential of TP-0903.

## Results

### TP-0903 reduces cell growth of NB cells but not non-tumor HK-2 cells

**Fig. 1:**
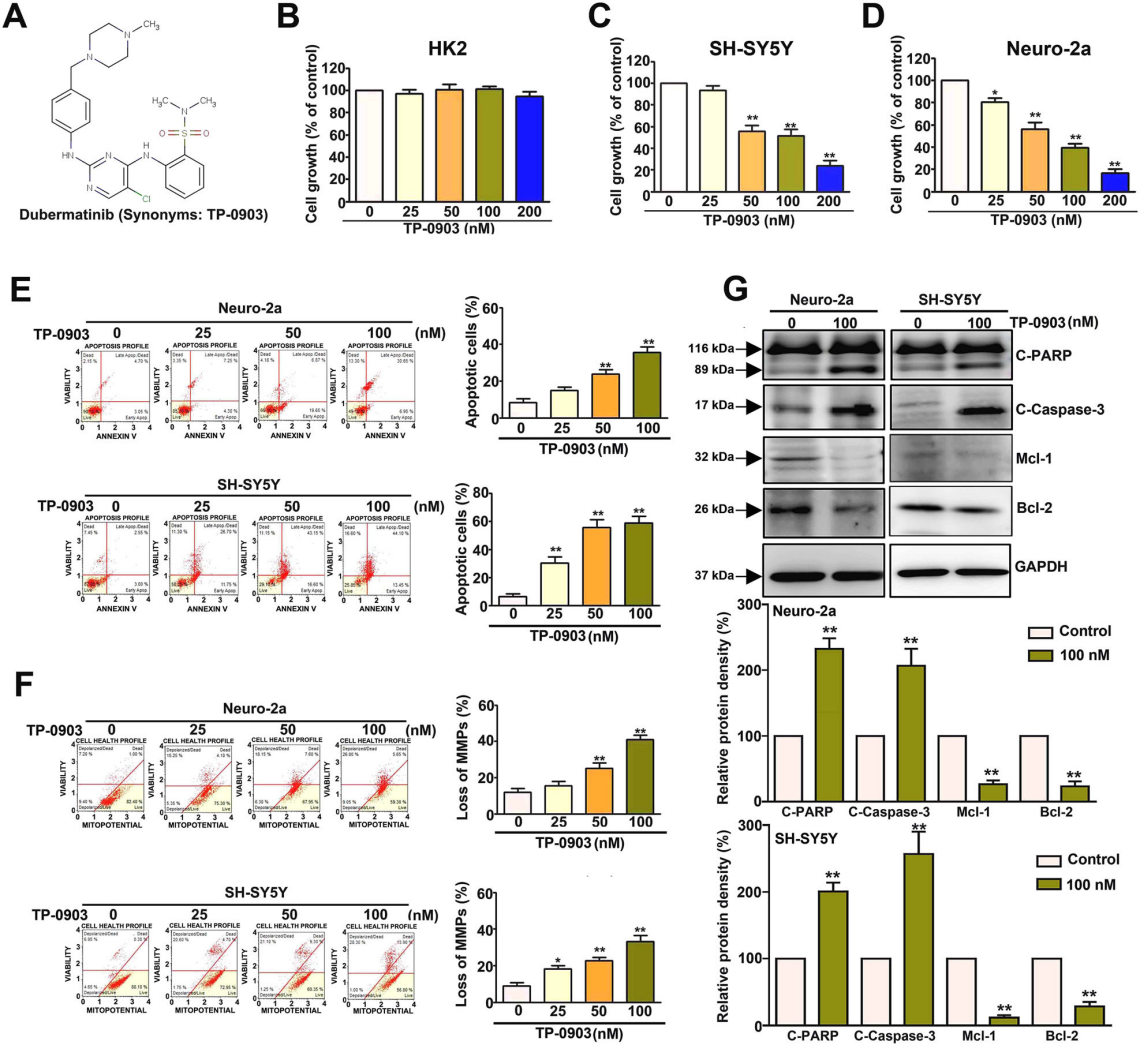
Effect of TP-0903 on cell growth, apoptosis and loss of mitochondrial membrane potentials of NB cells. **A** Structure of TP-0903. **B–D** Normal proximal tubular HK-2 and NB SH-SY5Y and Neuro-2a cells were treated with TP-0903 at serial concentrations for 24 h, and then the cell growth was determined using MTT assay. Cell growth was presented as percentage of control (0 μM, 100%). **E** Apoptosis assay using Annexin V/PI staining and flow cytometric analysis, (**F**) mitochondrial membrane potential (MMP) analysis, or (**G**) Western blot for apoptotic signaling cascade. Quantitative analysis of apoptotic cells or loss of MMP cells was presented as a percentage of total cells. Protein levels were semi-quantitated by densitometric analysis. GAPDH was used as internal control. * and **P < 0.05 and *P* < 0.01 as compared to control.

The chemical structure of TP-0903 (dubermatinib) is shown in Fig. 1A. The impacts of TP-0903 on the cell growth of non-tumoral tubular cell HK2 and NB cell SH-SY5Y and Neuro-2a. After 24 h treatments, TP-0903 (50–200 nM) significantly reduced the cell growth of SH-SY5Y and Neuro-2a cells in a dose-dependent manner (Fig. 1C, D). TP-0903 at 200 nM showed the maximal inhibitory effects and reduced the cell growth of SH-SY5Y and Neuro-2a cells to 21.2 ± 4.8% and 17.3 ± 3.1% of control, respectively (*P* < 0.01 vs control). Interestingly, TP-0903 did not influence the cell growth of HK2 cells (*P* > 0.3, Fig. 1B). Taken together, these findings indicate that TP-0903 inhibits the cell growth of NB cell SH-SY5Y and Neuro-2a cells, but insignificantly influences the cell growth of non-tumor HK2 cells.

### TP-0903 triggers cellular apoptosis and loss of mitochondrial membrane potential in NB cells

To further explore the inhibitory effects of TP-0903 on NB cells, apoptosis assay was then conducted. As shown in Fig. 1E, TP-0903 (50 ~ 100 nM) significantly triggered cellular apoptosis in Neuro-2a and SH-SY5Y cells in a dose-dependent fashion. TP-0903 at 100 nM contributed to the highest 58.3 ± 4.2% and 40.2 ± 2.4% cell apoptosis in Neuro-2a and SH-SY5Y cells, respectively (*P* < 0.05 vs control). TP-0903 is reported to induce cellular ROS production and mitochondrial apoptosis in breast cancer cells [10]. Next, the effect of TP-0903 on mitochondrial membrane potential (MMP) in NB cells is elucidated. As shown in Fig. 1F, TP-0903 led to the loss of MMP in Neuro-2a and SH-SY5Y cells in a dose-dependent manner. 100 nM TP-0903 caused the maximal loss of MMPs, which was 58.3 ± 4.2% and 40.2 ± 2.4% in Neuro-2a and SH-SY5Y cells, respectively. Based on the observations that TP-0903 induced cell apoptosis and loss of MMP, cellular apoptotic signaling was subsequently monitored. As shown in Fig. 1G, 100 nM TP-0903 treatment resulted in cleavage of PARP and caspase-3, while decreasing cellular level of anti-apoptotic Mcl-1 and Bcl-2 in both NB cells (*P* < 0.05 vs control). Collectively, these findings reveal that TP-0903 treatment induced apoptosis, MMP loss, and activation of apoptotic signaling, and downregulated anti-apoptotic Mcl-1 and Bcl-2 in NB cells.

### TP-0903 downregulates DKK1 expression, which is overexpressed in NB cells and tumors

**Fig. 2:**
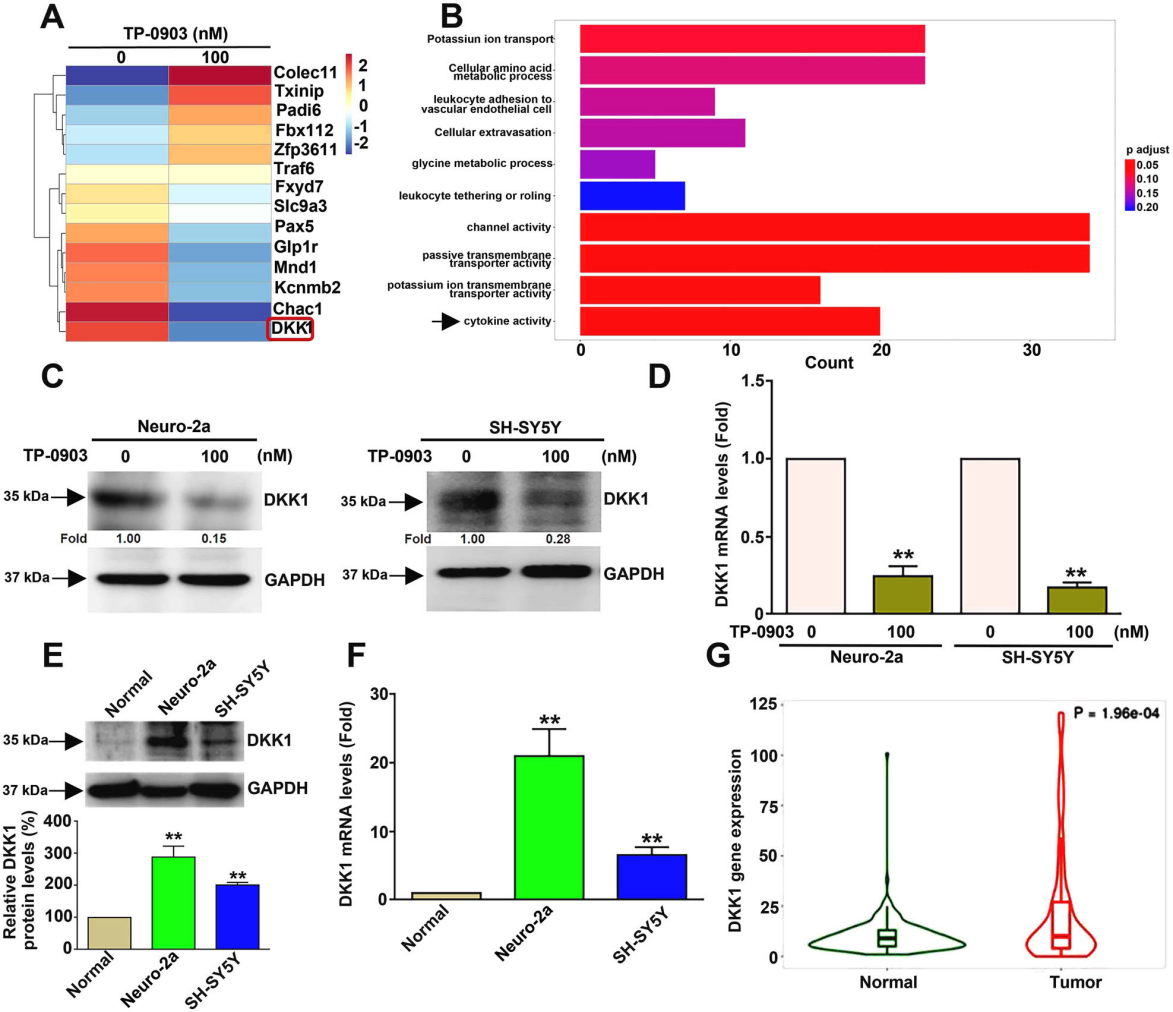
TP-0903 downregulates DKK1 and associated with cytokine activity pathway. Cells were treated with 100 nM TP-0903 for 24 h, and then subjected to total RNA extraction and gene expression analysis by RNA-seq analysis. **A** The heatmap of differentially expressed genes (DEGs) with -2≤ log_2_FC ≤ 2 in response to TP-0903 treatment. **B** The top 10 biological processes affected by the DEGs using Gene ontology analysis. *P*-values < 0.05 were indicated as red color. Cells were treated with 100 nM TP-0903 for 24 h, and then subjected to assessment of (**C**) protein levels or (**D**) mRNA expression levels by Western blotting or qRT-PCR analysis, respectively. **E** Protein levels and (**F**) mRNA expression levels of DKK1 among HK2 (Normal), Neuro-2a, and SH-SY5Y cells using Western blot and qRT-PCR, respectively. **P < 0.01 as compared to control. **G** Violin plots of DKK1 gene expression in paired normal (green) and NB tumor (red) gene array data from the TNMplot database.

To further explore the mechanistic action induced by TP-0903 in NB cells, systematic evaluation of differentially expressed genes (DEGs) in Neuro-2a cells in response to TP-0903 treatment was performed by using RNA-seq analysis. As shown in Fig. 2A, the heat map revealed a significant difference in gene expression between control and TP-0903 treatment. Among the DEGs induced by TP-0903 treatment, DKK1 was found to be significantly downregulated. Biological process analysis showed that the TP-0903-induced DEGs were involved in potassium ion transport, channel activity, passive membrane transporter activity, potassium ion membrane transporter activity, and cytokine activity (Fig. 2B, *P*-adjust <0.05). Consistent with RNA-seq analysis, DKK1 protein level and mRNA expression in NB cells were remarkably reduced by TP-0903 (Fig. 2C, D). In parallel, we assessed DKK1 protein level and mRNA expression in HK-2 cell (normal) and NB cells, and the results revealed that both protein and mRNA expression of DDK1 in NB cells were higher than HK-2 cells (Fig. 2E, F, *P* < 0.01). Next, the expression of DKK1 in NB tissues was elucidated by using TNM plot database analysis, and the results showed that DKK1 expression in NB tissues (tumor) was higher than normal tissues (Fig. 2G, *P* < 0.001). Together, these findings reveal that TP-0903 inhibits DKK1 expression which is highly expressed in NB cells and tumors.

### Involvement of DKK1 in TP-0903-triggered apoptosis of NB cells

**Fig. 3:**
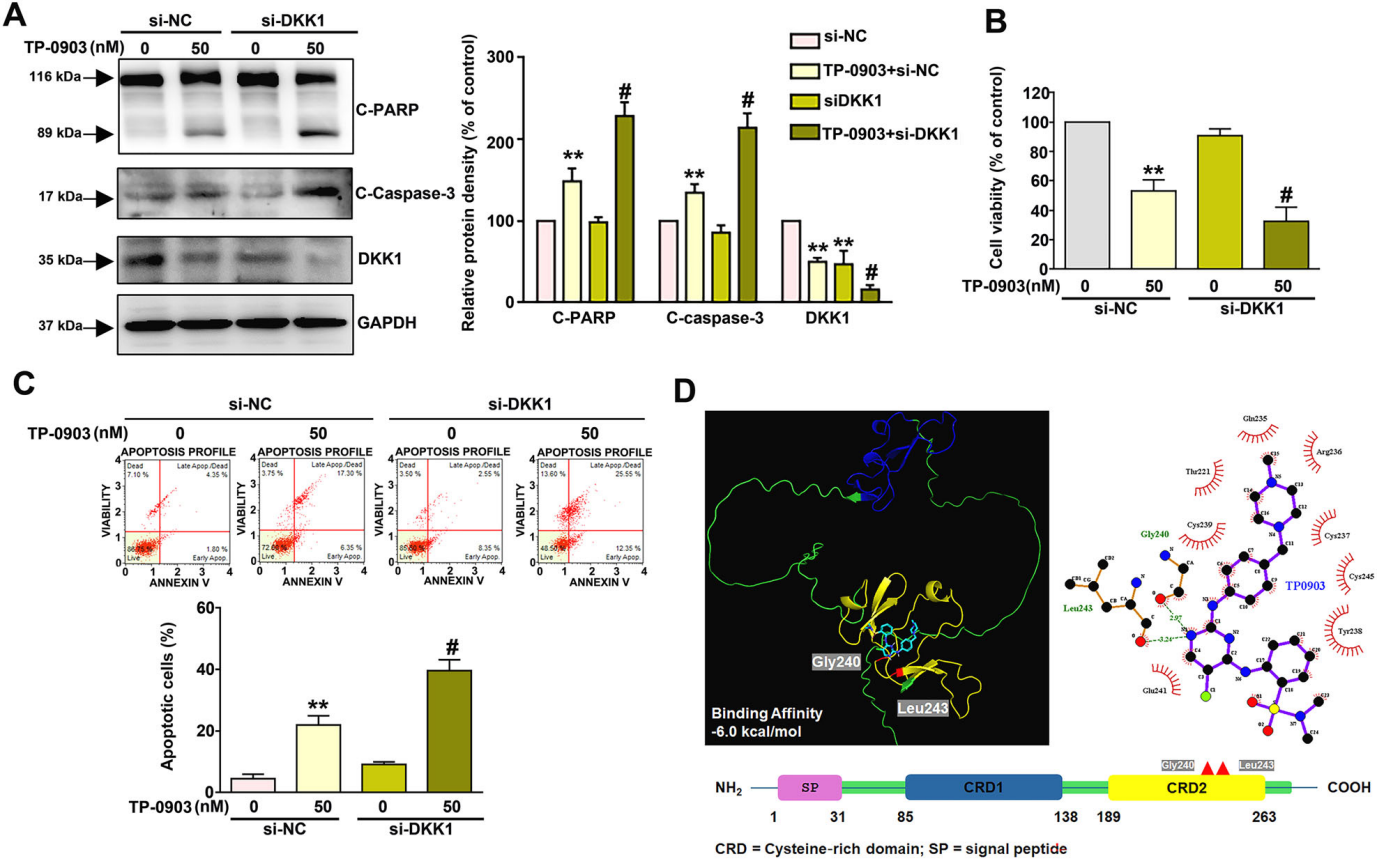
DKK1 knockdown promotes TP-0903-triggered cellular apoptosis and cell viability inhibition in Neuro-2a cells. Cells were transfected with siRNA against DKK1 (si-DKK1), treated with 50 nM TP-0903 for 24 h, and subjected to (**A**) immunodetection of cleaved PARP (C-PARP), cleaved caspase-3 (C-Caspase-3), and DKK1; (**B**) cell viability assay; or (**C**) cell apoptosis assay using Annexin V/PI staining and flow cytometric analysis. **P < 0.01 as compared to control (si-NC, 0 nM). ^#^*P* < 0.05 as compared to TP-0903 alone (50 nM). **D** Binding mode for docked ligand TP-0903 (cyan) was shown as stick representation in DKK1 (left panel). Structural domains of DKK1 and the amino acid residues interaction with TP-0903 were indicated. Binding affinity between TP-0903 and DKK1 was −6.0 kcal/mol.

Next, the involvement of DKK1 in TP-0903-induced apoptosis of NB cells was explored. As shown in Fig. 3A, TP-0903 induced cleavage (activation) of PARP and caspase-3, associating with downregulation of DKK1 in Neura-1a cells. Furthermore, the combination of TP-0903 and DKK1 knockdown via siRNA resulted in more PARP and caspase-3 cleavage than TP-0903 alone (*P* < 0.05). Consistently, the combination of TP-0903 and DKK1 knockdown also contributed to stronger viability inhibition (Fig. 3B) and apoptosis induction (Fig. 3C) in Neura-2a cells than TP-0903 alone (*P* < 0.05). Next, whether TP-0903 targets DKK1 protein was investigated using molecular docking analysis. The results show that DKK1 may interact with TP-0903 with a binding affinity of −6.0 kcal/mol (Fig. 3D, left panel), and the predicated interaction pattern reveals that that carboxyl group of Gly240 and Leu243 residue in DKK1 form a stable complex via strong hydrogen bond interactions with TP-0903 (Fig. 3D, right panel). Collectively, these findings reveal that DKK1 is involved in the TP-0903-induced apoptosis of NB cells, which may attribute to direct interaction with DKK1.

### TP-0903 upregulates miR-335-3p to reduce DKK1 expression and enhance apoptosis in NB cells

**Fig. 4:**
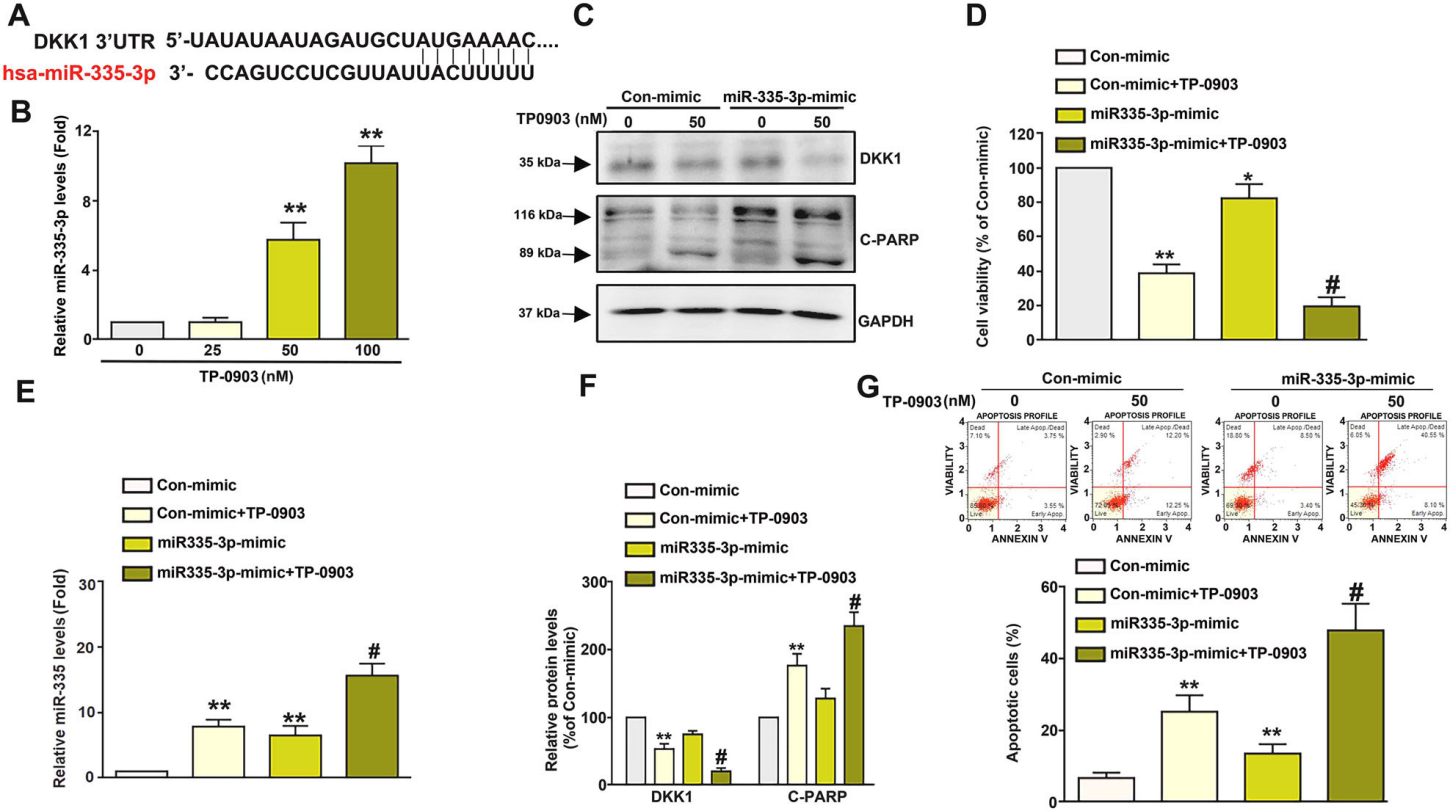
Involvement of miR-335-3p in TP-0903-triggered cellular apoptosis and downregulation of DKK1 in Neuro-2a cells. **A** Predicted binding site of miR-335-3p on 3’UTR of DKK1 gene. **B** Cells were treated with serial concentrations of TP-0903 for 24 h and subjected to assess miR-335-3p expression level by qRT-PCR. Neuro-2a cells were transfected with control-mimic or miR-335-mimic, treated with TP-0903, and subjected to (**C**) immunodetection of DKK1 and cleaved PARP (C-PARP) using Western blot analysis, (**D**) cell viability assay, (**E**) qRT-PCR to determine miR-335-3p expression level, (**F**) quantitation of signals on Western blots, or (**G**) cell apoptosis assay using Annexin V/PI staining and flow cytometric analysis. **P < 0.01 as compared to control. ^#^*P* < 0.05 as compared to TP-0903 alone (50 nM).

In parallel to possible interaction with DKK1, the role of miRNA in the downregulation of DDK1 by TP-0903 was further explored. As shown in Fig. 4A, bioinformatic analysis showed that has-miR-335-3p had a predicted binding to 46–52 of DKK1 3’ UTR. Therefore, expression level of miR-335-3p in Neuro-2a cells was assessed by using qRT-PCR, and the results revealed that TP-0903 significantly upregulated miR-335-3p expression (*P* < 0.005, Fig. 4B). Similarly, miR-335-3p mimics slightly reduced DKK1 protein level and induced PARP cleavage (P > 0.05, Fig. 4C, F). Notably, combination of TP-0903 with miR-335-3p mimics reduced the DKK1 protein level cleavage greater than TP-0903 alone. In addition, the combination also promoted PARP cleavage greater than TP-0903 alone (*P* < 0.05, Fig. 4C, F). Consistently with cellular apoptotic signals, miR-335-3p mimics moderately decreased viability of Neuro-2a cells (*P* < 0.05, Fig. 4D), and combination of TP-0903 with miR-335-3p mimics exhibited a greater viability inhibitory effect than TP-0903 or miR-335-3p mimics alone (*P* < 0.05, Fig. 4D). The similar effects of combination of TP-0903 with miR-335-3p mimics on apoptosis induction were also observed (Fig. 4G). Taken together, TP-0903 upregulates miR-335-3p, contributing to DKK 1downregulation and apoptosis induction.

### Involvement of cellular ROS in TP-0903-induced apoptosis, DKK1 downregulation, and miR335-3p upregulation in NB cells

**Fig. 5:**
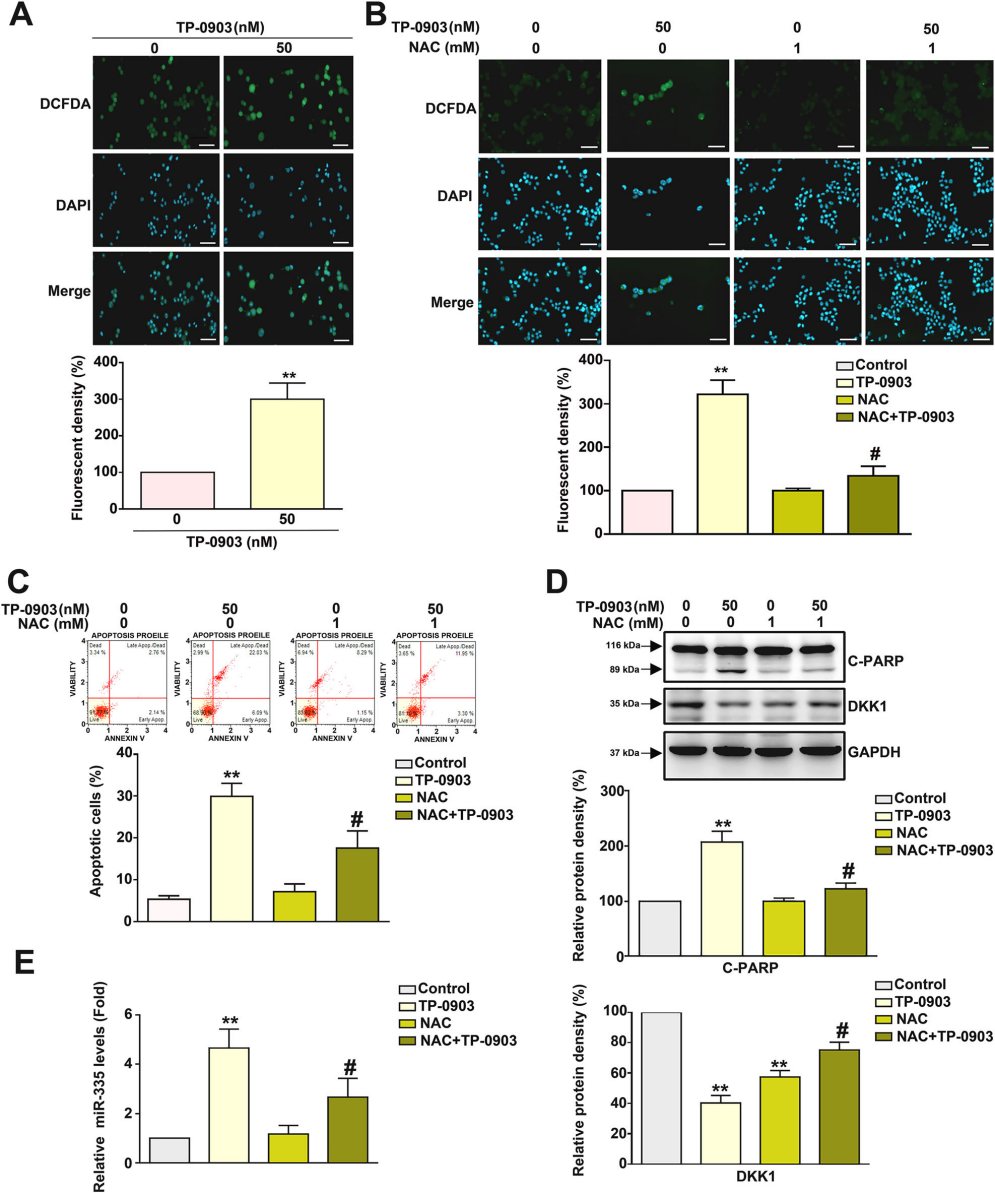
Involvement of ROS in TP-0903-induced apoptosis, miR-335-3p upregulation, and DKK1 downregulation in Neuro-2a cells. **A** Cells were treated with TP-0903 for 24 h and subjected to ROS assay. Cells were treated with 50 nM TP-0903 or TP-0903 combined with NAC for 24 h and subjected to (**B**) ROS assay, (**C**) apoptosis assay using Annexin V/PI staining and flow cytometric analysis, (**D**) immunodetection of C-PARP and DKK1 by Western blot, or (**E**) qRT-PCR to determine miR-335-3p expression level. GAPDH was used as internal control. Signals were semi-quantitated by densitometric analysis. **P < 0.01 as compared to control. ^#^*P* < 0.05 as compared to TP-0903 alone (50 nM). Scale bar=20 μm.

Elevated ROS play an important role in mitochondria dysfunction. Therefore, the involvement of ROS in TP-0903-mediated apoptotic cascades was elucidated. As shown in Fig. 5A, B, TP-0903 promoted cellular ROS generation in Neuro-2a cells, and the elevated cellular ROS was reduced by NAC cotreatment. Notably, NAC cotreatment also diminished TP-0903-induced cell apoptosis, PARP cleavage, and miR-335-3p expression, while increasing DKK1 expression (*P* < 0.05, Fig. 5C–E). Collectively, these findings reveal that cellular ROS is involved in TP-0903-induced apoptosis, DKK1 downregulation, and miR335-3p upregulation in NB cells.

### TP-0903 inhibits in vivo tumor growth and exhibits insignificant toxicity to organs in xenograft mouse model

**Fig. 6:**
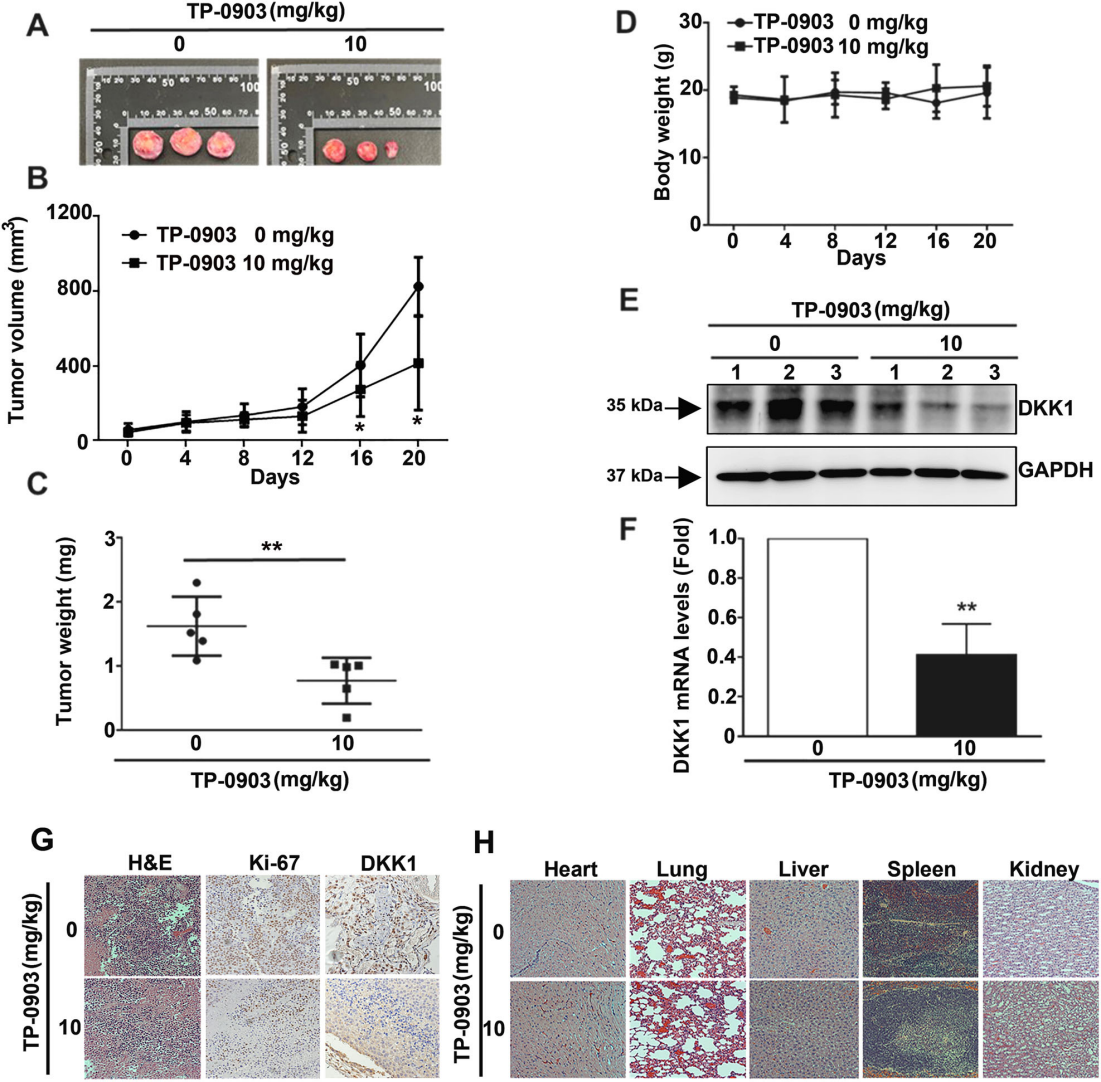
In vivo effects of TP-0903 on tumor growth using xenograft mouse model. Balb/c nude mice were inoculated with Neuro-2a cells, and then oral administration of PBS or TP-0903 (10 mg/kg) every 3 days for 20 days. **A** Representative tumors and (**C**) tumor weights in xenografted mice. Changes in (**B**) tumor volume and (**D**) mouse body weights during the 20-day treatment period. Tumors were acquired and subjected to (**E**) Western blot or (**F**) qRT-PCR to assess DKK1 protein and mRNA expression level, respectively. **G** IHC assay to assess cell proliferation and DKK1 expression in tumor tissues. **H** H&E staining to assess tissue morphology. * and **, *P* < 0.05 and *P* < 0.01 as compared to control group.

Next to the in vitro evaluation of anti-NB activity, in vivo effect of TP-0903 on NB growth was examined. As shown in Fig. 6A–C, the 20-day administration of TP-0903 (10 mg/kg) significantly inhibited the xenografted tumor volume and tumor weight in mice as compared to control (P < 0.01). In contrast to the inhibition of tumor growth, the body weight of xenograft mice was insignificantly influenced (*P* > 0.1, Fig. 6D). Consistently with the in vitro findings, Western blotting and qRT-PCR analysis revealed that TP-0903 administration significantly downregulated the protein level (Fig. 6E) and mRNA expression (Fig. 6F) of DKK1 in the tumor derived from inoculated Neuro-2a cells (*P* < 0.01). In addition to Western blotting and qRT-PCR analysis, the histological examination using H&E staining showed TP-0903 administration reduced the number of xenografted NB cells, and the IHC staining also revealed that the expression of cell proliferation marker Ki-67 and DKK1 were decreased in tumor tissues in response to TP-0903 administration (Fig. 6G). Furthermore, the effect of TP-0903 administration on important organs in xenograft mice was investigated. Our observations showed that TP-0903 administration did not significantly affect the histological features of heart, lung, liver, spleen, and kidney tissues (Fig. 6H). Collectively, these in vivo findings show that TP-0903 reduces the growth of tumor derived from Neuro-2a cells and inhibits DKK1 expression, but insignificantly affects the body weight and histological feature of organs.

## Discussion

**Fig. 7:**
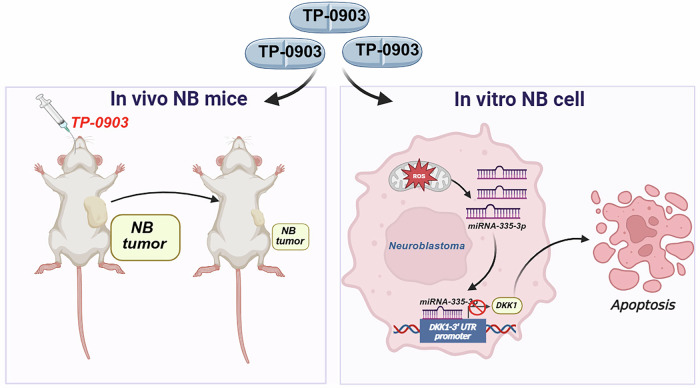
Proposed mechanism for anticancer effect of TP-0903 on NB cells. TP-0903 promotes ROS generation, upregulates miR-335-3p expression, consequently downregulating DKK1 expression and leading to cell apoptosis.

TP-0903 was developed as a type I inhibitor specifically targeting the AXL receptor tyrosine kinase, producing irreversible cytotoxic effects and triggering caspase-dependent apoptosis. Additionally, TP-0903 disrupted both colony formation and neurosphere generation necessary for neuroblastoma cell expansion, thereby increasing their sensitivity to standard chemotherapy agents [12]. In this study, we unveil that TP-0903 significantly suppresses viability of NB cells and induces apoptosis, attributing to promoted ROS-mediated miR-335-3p upregulation and the subsequent downregulation of DKK1 (Fig. 7). These results demonstrate a previously unknown anticancer activity of TP-0903 against neuroblastoma and extend our knowledge of the anti-neuroblastoma therapeutic potential of the ROS/miR-335-3p/DKK1 axis.

The intrinsic apoptotic pathway represents an attractive therapeutic target, as neuroblastomas frequently evade apoptosis through increased expression of the anti-apoptotic protein Bcl-2 [20, 21]. The selective Bcl-2 inhibitor venetoclax has demonstrated encouraging effectiveness in laboratory and animal studies and is currently undergoing pediatric phase I clinical evaluation (NCT03236857); however, preclinical research indicates that monotherapy with venetoclax typically fails to achieve complete tumor elimination due to treatment-induced resistance mechanisms. This resistance occurs through elevated expression of another anti-apoptotic protein, Mcl-1, which sequesters Bim that has been released from Bcl-2 by venetoclax, creating an alternative apoptosis evasion mechanism. Similarly, monotherapy with Mcl-1 inhibitors promote resistance via upregulation of other anti-apoptotic Bcl-2 family such as Bcl-2 and Bcl-xL [22, 23]. Our results show that TP-0903 both decreases Bcl-2 and Mcl-1 levels in NB cells, implicating that TP-0903 not only triggers apoptosis but also reduces apoptosis evasion in NB treatment.

The DKK1 is a member of the Dickkopf gene family, which comprises Dickkopf-1, -2, -3 and -4, and Dickkopf-3-associated protein, Soggy [24]. DKK1 is an important regulator of the Wnt/β-catenin signaling pathway, which mediates a spectrum of cellular processes, including cell survival, proliferation, apoptosis and motion [25]. DKK1 overexpression has been observed in various cancers such as esophageal carcinoma, lung cancer, breast cancer, and liver cancer [15, 26, 27]. Moreover, in oxaliplatin-resistant colon cancer cells, DKK1 signaling is remarkably increased that contributes to AKT activation, and suppression of DKK1 signaling significantly promotes the sensitivity to oxaliplatin, suggesting that DKK1 functions as a chemoresistant factor in colon cancer through enhancing AKT activation [28]. A meta-analysis also demonstrated that increased DKK1 expression correlates with shorter progression-free survival, disease-free survival, and time to recurrence in various cancers, including digestive system cancers [29]. Similarly, our analyses reveal that DKK1 exhibits a higher level in NB tumor tissues than normal neuronal tissues, as well as a higher gene expression in Neuro-2a and SH-SY5Y cells than HK2 cells. Furthermore, we demonstrate that potential direct binding between TP-0903 and DKK1 and TP-0903 combined with DKK1 knockdown provokes a greater apoptosis of NB cells than TP-0903 alone. Collectively, these observations implicate that DKK1 play a role in apoptosis resistance in NB and that TP-0903 may hinder DKK1 through direct interaction. It suggests that DKK1 is a potential target for NB treatment.

miRNA is a key regulator for gene expression, and mutations or reduced expression of miRNAs are highly associated with malignant tumors [30, 31]. Mounting evidences have shown that the restoration of tumor suppressor miRNAs and the inhibition of oncogenic miRNAs are now crucial potential targets for cancer therapy [32]. In osteoblast, miR-335-3p directly targeting DKK1 mRNA through complementary binding to its 3’-untranslated region (3’UTR) [19]. Our analysis revealed the presence of a potential miR-335-3p binding region in the 3’UTR of DKK1 and demonstrated that TP-0903 upregulated miR-335-3p expression in Neuro-2a cells in a dose-dependent manner. Moreover, miR-335-3p mimics combined with TP-0903 synergistically decreases Neuro-2a cell viability and provokes apoptosis (Fig. 5D, G, *P* < 0.05 vs control), implicating that miR-335-3p is involved in governing NB cell survival and apoptosis. Interestingly, miR-335-3p mimics alone reduced cell viability and promoted cell apoptosis of Neuro-2a cells, but insignificantly affected the cleaved PARP level (Fig. 5C–G). Furthermore, TP-0903 alone induced more apoptosis in Neuro-2a cells compared with the miR-335-3p analog alone. Taken together, these observations suggest that TP-0903 promotes apoptosis of NB cells attributing to not only upregulation of miR-335-3p but also other apoptotic machinery. miR-493-3p has been reported to inhibit growth of gastric cancer cells via downregulation of DKK1 [33]; nevertheless, the role and relationships of miR-493-3p and DKK1 in NB is rarely explored. Furthermore, whether TP-0903 affects miR-493-3p or other miRNAs that may target DKK1 and their roles in TP-0903-induced apoptosis in NB cells still need more investigation.

ROS is a key regulator in modulating multiple cellular processes and signaling pathways. Previous studies have shown that upregulation of AXL/GAS6 ameliorates the hypoxia-induced ROS caused apoptosis of NB cells [34, 35], indicating that AXL/GAS6 plays a crucial role in reducing oxidative stress and its associated cell damage and apoptosis. Our results showed that the AXL inhibitor TP-0903 significantly induced cellular ROS generation, further highlighting the role of AXL in regulating cellular ROS. Moreover, our findings demonstrate the TP-0903-induced ROS upregulates miR-335-3p, subsequently downregulating DKK1, and promoting apoptosis in NB cells. These observations suggest that ROS induced by AXL inhibition modulates the expression of miRNAs and their target genes, leading to NB cell death. Notably, reduction of ROS by NAC did not completely reverse the upregulation of miR-335-3p, downregulation of DKK1, and cell apoptosis in response to TP-0903 treatment (Fig. 6). It implicates that TP-0903 modulates these cellular processes through multiple mechanisms other than ROS. However, it requires further investigation to elucidate.

In this study, we also find that DKK1 is upregulated in and in NB tumors, and demonstrate that TP-0903 remarkably reduces gene expression and protein level of DKK1 in NB cells, attributing to upregulation of cellular ROS and miR-335-3p and potential binding of TP-0903 to DKK1. Furthermore. our findings indicate that DKK1 suppression potentiates apoptosis and inhibits viability of NB cells. These findings suggest that DKK1 inhibition is a potential therapeutic target for NB treatment.

## Conclusion

Conclusively, our results demonstrate that TP-0903 significantly inhibits viability and triggers apoptosis of NB cells and suppresses in vivo tumor growth derived from NB cells. The inhibitory effect of TP-0903 on NB cells is attributed to excessive ROS generation, upregulation of miR-335-3p, and downregulation of DKK1. These findings not only demonstrate that TP-0903 has a potent antiproliferative potential against NB cells, but also underline the significance of targeting miR-335-3p/DKK1 axis for NB therapy.

## Materials and methods

### Chemicals and antibodies

TP-0903 (HY-12963) was purchased from MedChemExpress. Chemicals and reagents without specific indication were purchased from Sigma-Aldrich (St. Louis, MO, USA). Antibodies binding to cleaved poly(ADP-ribose) polymerase (C-PARP; #9542, Cell Signaling Technology), cleaved caspase-3 (C-caspase-3; sc-56053, Santa Cruz Biotechnology), Mcl-1 (SC-12756, Santa Cruz Biotechnology), Bcl-2 (A11025, ABclonal, Inc), Dickkopf-1 (DKK1; AB61034, Abcam), glyceraldehyde 3-phosphate dehydrogenase (60004-1-Ig, Proteintech Group, Inc), and peroxidase-conjugated secondary anti-mouse IgG (sc-2005; Santa Cruz Biotechnology). The hsa-miR-335-3p mimic or control mimic were purchased from TrustGene Biotech company.

### Cell culture

Mouse NB cell Neuro-2a (BCRC # 60026) and human kidney epithelial cell HK2 (BCRC #60097) were obtained from BCRC (Hsinchu, Taiwan). Human NB cell SH-SY5Y (ATCC #CRL-2266) were obtained from ATCC (Rockville, MD, USA). Neuro-2a and SH-SY5Y cells were maintained in MEM supplemented with glutamine, NaHCO_3_, NEAA, sodium pyruvate (Gibco), and 10% fetal bovine serum (FBS, Hyclone). HK2 cells were cultured in keratinocyte-serum free medium supplemented with 5 ng/mL recombinant epidermal growth factor. Cells were grown at 37 ^o^C in a 5% CO_2_ -controlled humidified incubator. Cells reached 70-80% confluency were treatment with TP0903 or siRNA.

### Cell growth assay

Cell growth was assessed using MTT assay as previously described [36]. Briefly, cells were seeded into a 6-well plate (1 × 10^4^ /well) and treated with TP-0903 at 25, 50, 100, and 200 nM for 24 hours (h), and then reacted with the MTT solution for 4 h. Then the absorbance of the solution at 563 nm was determined using a spectrophotometer. The cell growth was presented as percentage of control treatment.

### Cell apoptosis and mitochondrial membrane potential analysis (MMPs) assessment

Cell apoptosis was assessed as previously describe [36]. Briefly, cells were washed with PBS, collected by spin-down, fixed with ethanol, and then stained with Muse® Annexin V & Dead Cell Kit (apoptosis assay) or MitoPotential reagent (MMPs) for 15 min and then subjected to flow cytometry analysis using a Muse cell analyzer (Luminex Corporate). Each result was acquired from 10,000 cell counts.

### Immunoblotting evaluation

Protein extraction and immunoblot were performed as previously described [37]. Briefly, cell lysis was performed using RIPA buffer containing protease and phosphatase inhibitor cocktail (Sigma-Aldrich). After removing insoluble debris, the crude proteins in extracts were quantified by Bradford method and separated by 10–12% SDS-PAGE. Then, the proteins were transferred onto a polyvinylidene difluoride membrane (Immobilon, Merck, Darmstadt, Germany), and probed with specific antibodies for detection of target proteins. Signals was developed using an ECL chemiluminescence reagent and semi-quantified using an ImageQuant LAS 4000 Mini imaging system (GE Healthcare). Signals of GAPDH were served as internal control.

### RNA sequencing analysis

Cells were treated with 100 nM TP-0903 for 24 h, then subjected to total RNA extraction. Extracted RNA quality was evaluated using an Agilent 2100 Bioanalyzer. The RNA sequencing analysis was performed by BIOTOOLS Co., Ltd., New Taipei, Taiwan. The purified mRNA was fragmented, and pair-end RNA sequencing was carried out using the Illumina HiSeq platform. Differential gene expression between TP-0903 and control groups was assessed with DESeq2 v1.34.021 package. Gene expression was normalized with the median of ratios method from DESeq2. Differentially expressed genes (DEGs) were defined as genes with false discovery rates (FDR) < 0.01 and subjected to Gene ontology (GO)/Biological process analysis.

### TNMplot analysis

DKK1 gene expression in NB tumor and normal samples was compared by using TNMplot analysis (http://www.tnmplot.com) as previously described [38]. The results were visualized by violin plots, and P value for the comparison was indicated.

### qRT-PCR

For DKK1 mRNA expression, total RNA was extracted by Trizol (Invitrogen), and the complementary DNA (cDNA) was obtained by reverse transcription using the ReverTra Ace qPCR RT Master Mix kit (TOYOBO, Japan). The qPCR was conducted by using a StepOne Real-Time PCR System (Applied Biosystems, Foster City, USA). The primers used for qRT-PCR included: DKK1, (F) 5’-GGTCCCGAAGTTGAGGTTCC-3’, (R) 5’-CATCATCTCCGAAGGACGCA-3’; GAPDH. (F) 5’‑AGGTCGGTGTGAACGGATTTG‑3’, (R) 5’‑TGT AGA CTP-0903 TGT AGT TGA GGT. Relative gene expression quantitation was normalized to endogenous GAPDH using the 2^-ΔΔCt^ method. The miR-335-3p expression was evaluated by qPCR using a specific primer UCAAGAGCAAUAACGAAAAAUGU and AUUUUUCGUUAUUGCUCUUGAUU.

### DKK1 silencing by small inhibitory RNA (siRNA)

DKK1 expression silencing was achieved by using specific small inhibitory RNAs (siRNAs) according to the manufacturer’s instructions. Briefly, Neuro-2 cells were transfected with the DKK1 siRNA (sc-37083, Santa Cruz Biotechnology) using RNAiMAX Transfection Reagent (Thermo Fisher Scientific, Waltham, MA, USA) at 37 °C and 5% CO_2_ for 48 h. The mouse Dkk-1 siRNA is a pool of 3 different siRNA duplexes, sc-37083A: sense: GAACCACACUGACUUCAAAtt, antisense: UUUGAAGUCAGUGUGGUUCtt; sc-37083B, sense: CAAGCCAGCAAUUCUUCUAtt, antisense: UAGAAGAAUUGCUGGCUUGtt; sc-37083C: sense: GUACACGGUUGAUUGUCUUtt, antisense: AAGACAAUCAACCGUGUACtt.

### Molecular docking for molecular interaction

Auto Dock Vina was utilized for the molecular docking analysis of TP-0903 with DKK1 using a flexible docking protocol as previously described [39]. The structure of TP-0903 was acquired from NCBI Pubchem (CID: 56839178). Previously created PDB file was used as input to generate the protein data bank, partial charge, and atom type (PDBQT) files. The other parameters were kept as default, and all bonds contained in ligand were set freely to rotate. The most suitable docking pose which exhibited the lowest binding energy (kcal/mol) and root mean square deviation conformation was presented. The interaction between TP-0903 and DKK1 was prepared, visualized, and analyzed using PyMOL and Discovery Studio 2016, respectively.

### Transfection of hsa-miR-335-3p mimic

Cells were transfected with 50 nM hsa-miR-335-3p mimic or control mimic with RNAiMAX Transfection Reagent (Thermo Fisher Scientific, Waltham, MA, USA) according to the manufacturer’s instructions.

### Assessment of cellular ROS

Cells were cultured in 12-well plates and treated with TP-0903 for 24 h. Then, the cellular ROS levels were assessed by using DCFH-DA assay as previous study [40]. Briefly, cells were stained with 10 µM DCFH-DA for 30 min in dark, and then the ROS was visualized by using the ImageXpress Pico automated cell imaging system to observe the fluorescence corresponding to ROS in the cells were captured. The images were then analyzed in Image-J software for the determination of changes in ROS.

### In vivo xenograft mouse model

Female 5-week-old Balb/c nude mice were acquired from the National Institutes of Applied Research National Center for Biomodels (Taipei City, Taiwan) and maintained in individually ventilated cages under s specific pathogen-free conditions under the performed in accordance with the Institutional Animal Ethical Guidelines of Chung Shan Medical University. We use five animals per group to provide reliable and reproducible results while achieving to the 3 R principles (Replacement, Reduction, and Refinement) in animal research. All mice were randomly assigned to receive subcutaneous injections of Neura-2a cells (2 × 10⁶) into nude mice. Five mice were divided into two experimental groups: control group and TP-0903 (10 mg/kg) group. After tumor size reached approximately 100 mm^3^ (day 0) and began oral administration of TP-0903 (10 mg/kg) once every three days for 20 days. Body weight of each mouse was measured every four days to monitor the toxicity of TP-0903. All the mice were sacrificed at the end of treatment, and the tumors were excised and subjected to volume and weight measurements, histological examination with H&E staining, and immunohistochemical analysis for Ki-67 (cell proliferation marker) and DKK1. Organ toxicity was assessed by using histological examination for heart, lung, liver, spleen, and kidney. The in vivo experimental procedure was designed to minimize animal use and reduce animal suffering.

### Statistical analysis

Quantitative data from three independent experiments were used for statistical analysis and presented as the mean ± standard deviation (SD). Student’s *t* test was used to analyze the significance of difference between two treatments and the one-way analysis of variance (ANOVA) by Dunnett test was conducted for the different treatment group. *P* < 0.05 or *P* < 0.01 were considered as significant.

## Supplementary information

Original data

## Data Availability

The data that support the findings of this study are available from the corresponding author upon reasonable request.
