# Supplementary figures and images for: AXL tyrosine kinase inhibitor TP-0903 induces ROS trigger neuroblastoma cell apoptosis via targeting the miR-335-3p/DKK1 expression
Source: Cell Death Discov. 2025 Aug 13;11:378. doi: 10.1038/s41420-025-02681-9 (PMC12350951; doi:10.1038/s41420-025-02681-9)

Fig-1G

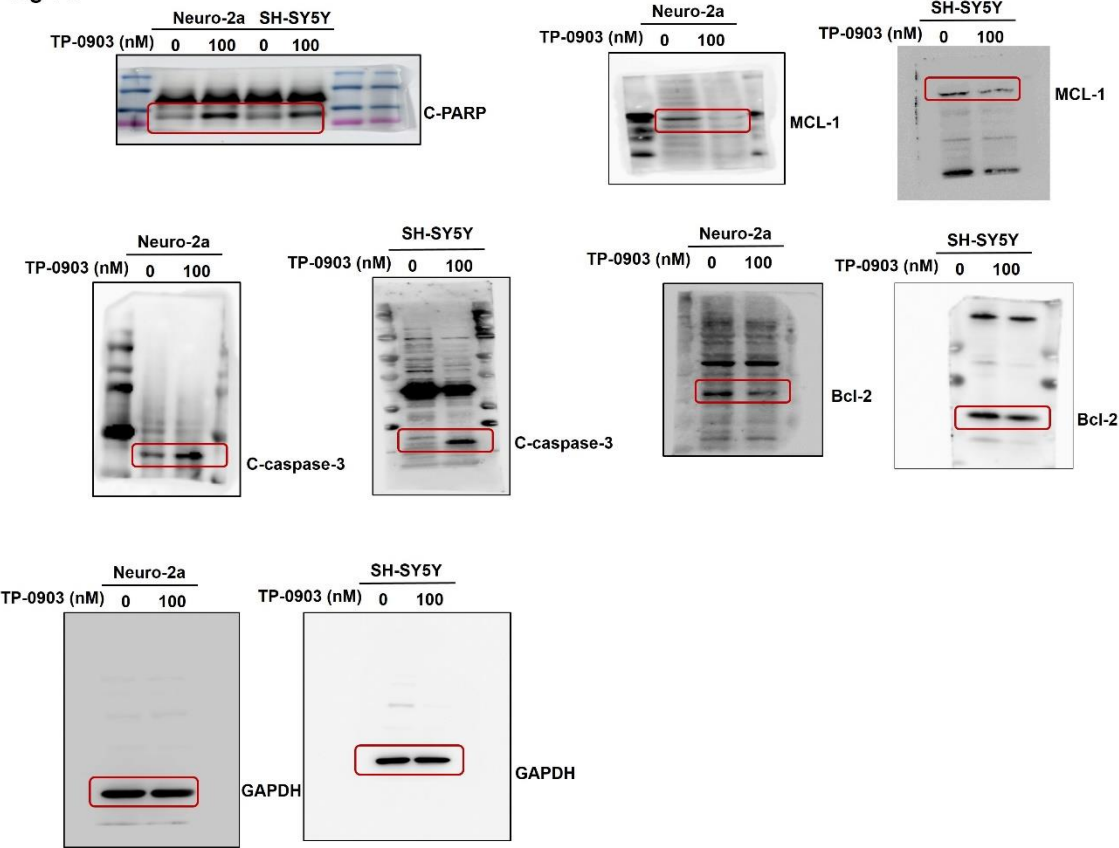

**Fig-2C**

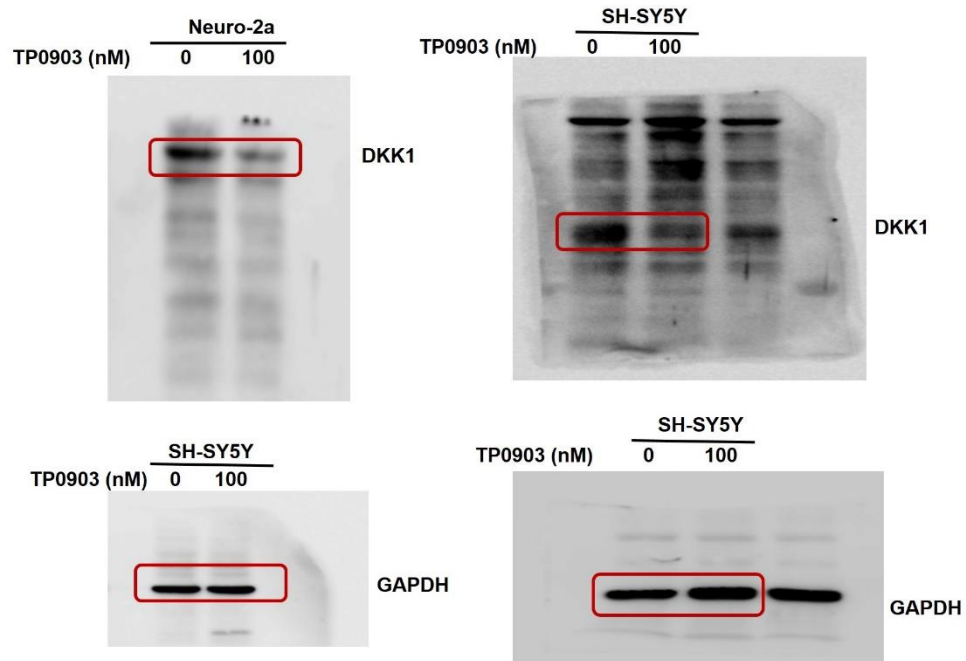

**Fig-2E**

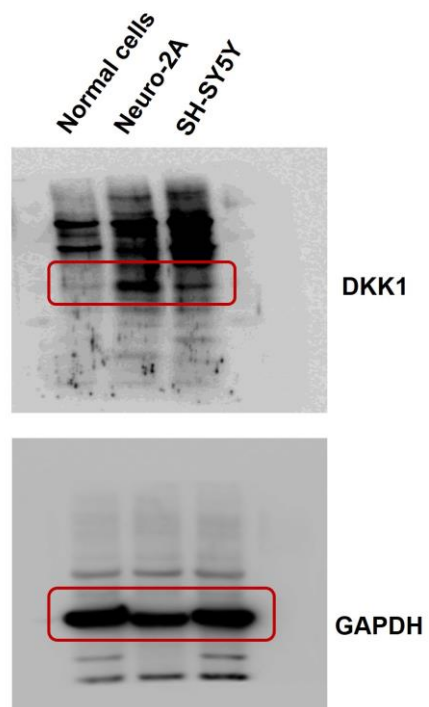

**Fig-3A**

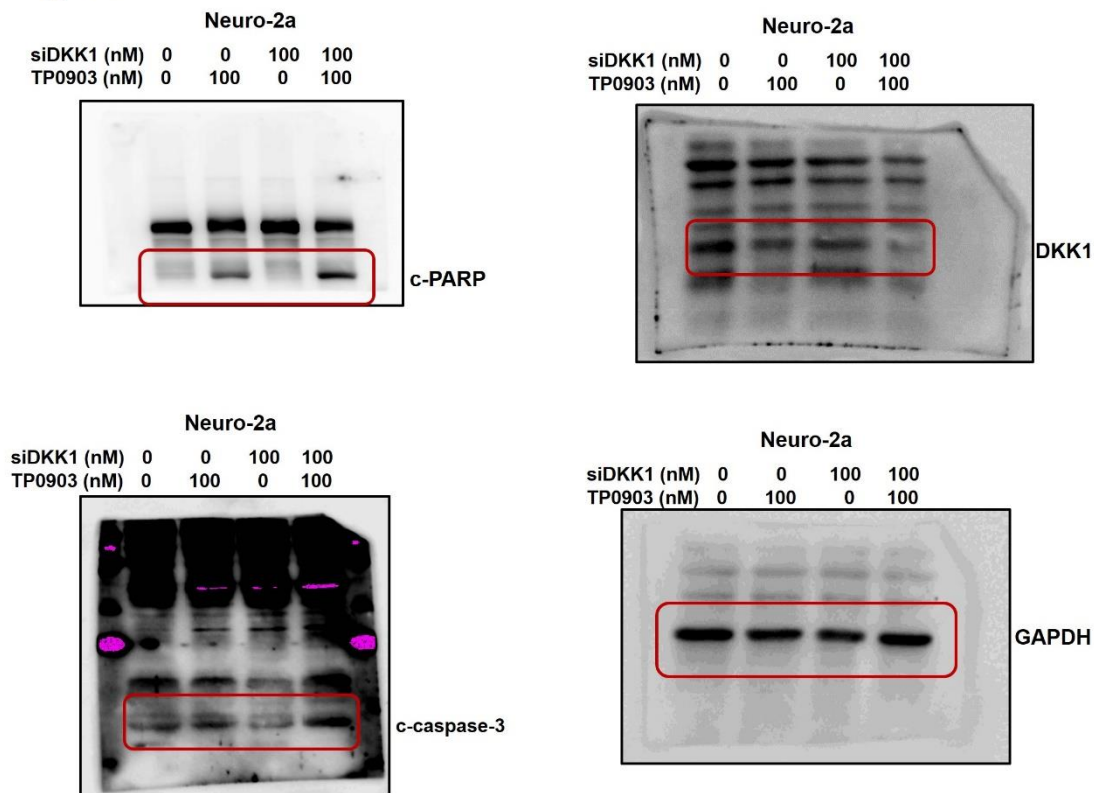

**Fig-4C**

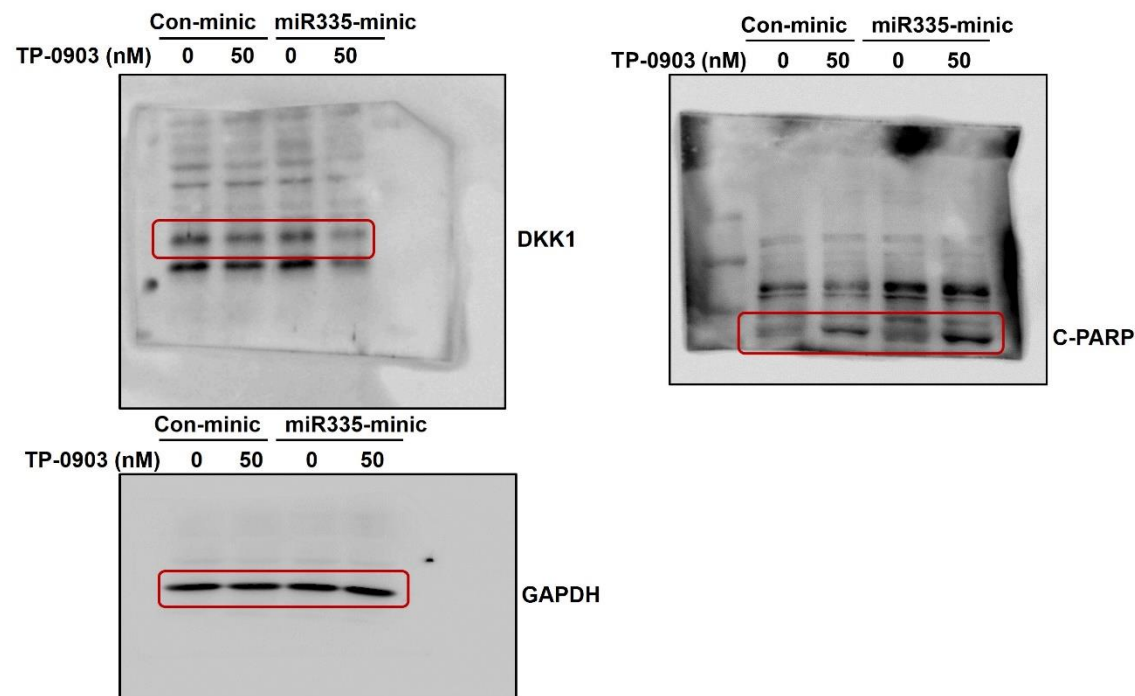

**Fig-5D**

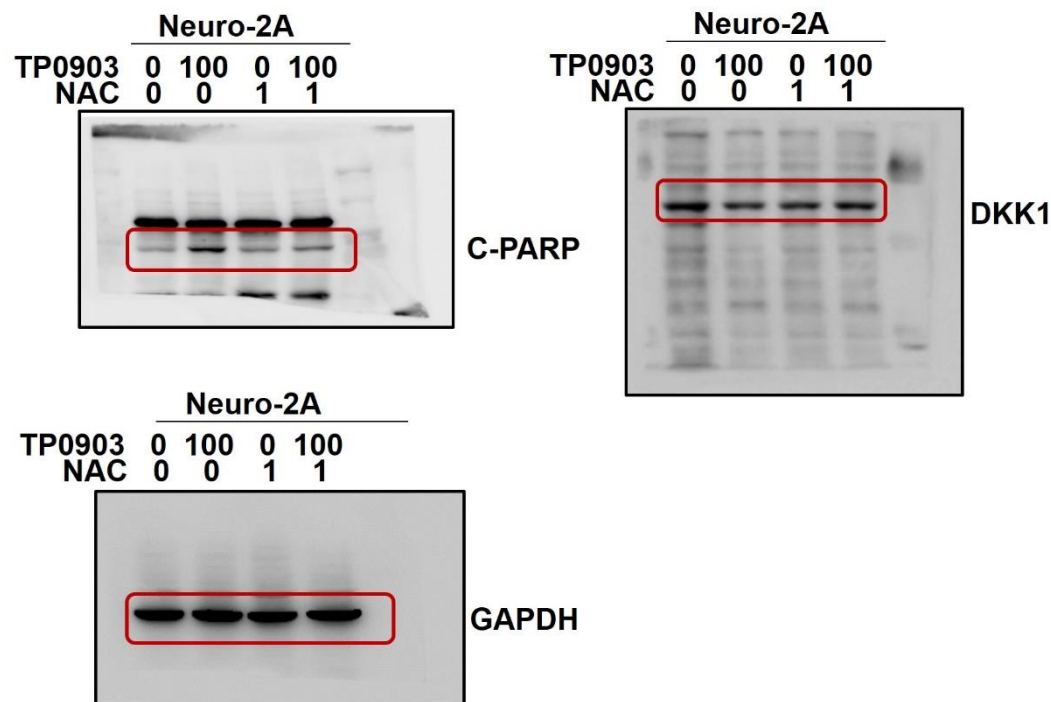

**Fig-6E**

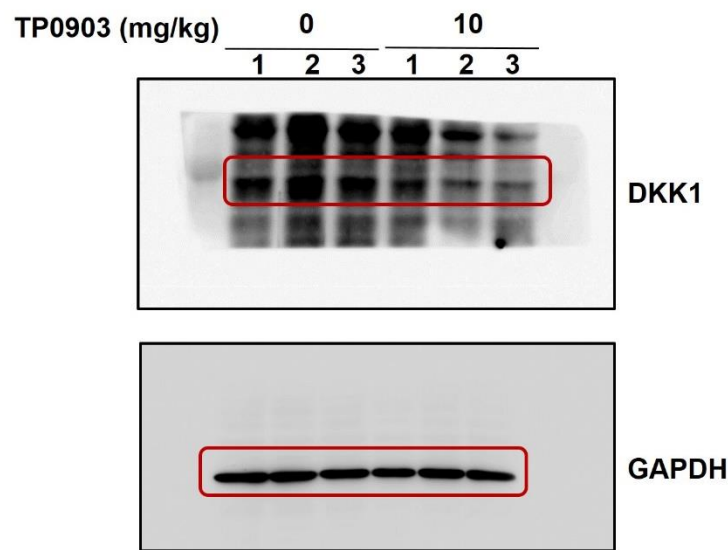

Supplement: Supplementary file 1 — Original data [file 41420_2025_2681_MOESM1_ESM.pdf]
